# Dipyrazolo[1,5-*a*:4',3'-*c*]pyridines – a new heterocyclic system accessed via multicomponent reaction

**DOI:** 10.3762/bjoc.8.251

**Published:** 2012-12-27

**Authors:** Wolfgang Holzer, Gytė Vilkauskaitė, Eglė Arbačiauskienė, Algirdas Šačkus

**Affiliations:** 1 Department of Drug and Natural Product Synthesis, Faculty of Life Sciences, University of Vienna, Althanstrasse 14, A-1090 Vienna, Austria; 2 Institute of Synthetic Chemistry, Kaunas University of Technology, Radvilėnų pl. 19, 50254 Kaunas, Lithuania

**Keywords:** cyclization, nitrogen heterocycles, NMR spectroscopy, multicomponent reaction, pyrazole

## Abstract

The synthesis of dipyrazolo[1,5-*a*:4',3'-*c*]pyridines is described. Easily obtainable 5-alkynylpyrazole-4-carbaldehydes, *p*-toluenesulfonyl hydrazide, and an aldehyde or ketone containing an α-hydrogen atom were reacted in a silver triflate catalyzed multicomponent reaction affording new tricyclic compounds with a dipyrazolo[1,5-*a*:4',3'-*c*]pyridine core. Detailed NMR spectroscopic investigations (^1^H, ^13^C and ^15^N) were undertaken with all obtained compounds.

## Introduction

Condensed pyrazole scaffolds are important substructures of compounds with biological activity and can be found in some well-known drug molecules, such as, for example, Sildenafil (a pyrazolo[4,3-*d*]pyrimidine) [1–2], Allopurinol (a pyrazolo[3,4-*d*]pyrimidin-4-one) [3], Zaleplon (a pyrazolo[1,5-*a*]pyrimidine) [4], and Zolazepam (a pyrazolo[3,4-*e*][1,4]diazepine derivative, used in veterinary medicine) [5]. Particularly pyrazolopyrimidines are a very frequently accessed class of compounds [6] with the particular importance of the pyrazolo[3,4-*d*]pyrimidine core, which can closely mimic the purine system of adenosine and, thus, has been incorporated in various compounds impairing protein kinases and ATPases [7–9]. Moreover, a large variety of additional fused pyrazoles exhibit interesting biological activities, such as, pyrazolo[1,5-*a*]quinolones [10], pyrazolo[4,3-*c*]quinolones [11], pyrazolo[5,1-*a*]isoquinolines [12], and thieno[2,3-*c*]pyrazoles [13]. In view of these facts as well as due to our continuing interest in the exploration of useful but unused chemical space, which has become a paradigm of contemporary medicinal chemistry [14], we have devoted some effort to the construction of novel condensed heterocyclic systems containing pyrazole substructures. Apart from a series of publications dealing with the construction of pyrazole analogues of xanthones and related systems [15–20] we recently described the synthesis of pyrano[4,3-*c*]pyrazol-4(1*H*)-ones and -4(2*H*)-ones [21], 1,5-dihydro- resp. 2,5-dihydro-4*H*-pyrazolo[4,3-*c*]pyridin-4-ones [20], and pyrazolo[4,3-*c*]pyridines [22]. In continuation of these studies we herein present the synthesis and detailed NMR spectroscopic characterization of the new heterocyclic system dipyrazolo[1,5-*a*:4',3'-*c*]pyridine. The access to the latter was achieved by a multicomponent reaction (MCR) starting from 5-alkynylpyrazole-4-carbaldehydes. Such MCRs, although being present since the early days of organic chemistry, nowadays attract an increasing interest because of their unmatched synthetic efficiency, which permits the construction of complex molecules in an elegant and sufficient manner [23–26].

## Results and Discussion

### Chemistry

The starting compounds for the construction of the title compounds are the 5-alkynylpyrazole-4-carbaldehydes **1**. Their synthesis from easily accessible 2-pyrazolin-5-ones through Vilsmeier formylation (with concomitant transformation of the oxygen into a chlorine substituent), followed by Sonogashira cross-coupling reaction of the obtained 5-chloropyrazole-4-carbaldehydes with appropriate alkynes, has been described by us in a former publication [21]. Compounds **1** containing an alkyne function and a nucleophilic substituent in the *ortho* position of the pyrazole system are valuable precursors for the construction of corresponding annulated systems. In order to employ this arrangement of functionalities for the latter purpose, we adapted the approach of Wu and co-workers, who described the synthesis of *H*-pyrazolo[5,1-*a*]isoquinolines by a one-pot tandem reaction of 2-alkynylbenzaldehydes, sulfonohydrazide, and ketones or aldehydes [27]. In our case, application of pyrazolecarbaldehydes **1** should enable access to the desired dipyrazolo[1,5-*a*:4',3'-*c*]pyridines **5**.

**Scheme 1:**
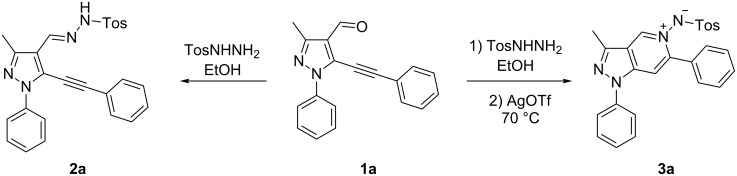
Intermediate reactions of pyrazole-4-carbaldehyde **1a**.

In order to test the reaction conditions, firstly each step of the multicomponent reaction was carried out separately. According to the strategy, pyrazole-4-carbaldehyde **1a** was primarily condensed with *p*-toluenesulfonyl hydrazide affording hydrazide **2a** in 97% yield (Scheme 1). Secondly, two steps of the applied strategy were performed at once: condensation of **1a** with *p*-toluenesulfonyl hydrazide and subsequent 6-*endo*-*dig* cyclization [28] of intermediate **2a** in the presence of silver triflate produced *p*-toluenesulfonylazamide **3a** in 91% yield (Scheme 1). The reaction of intermediate **3a** with CH-acidic aldehydes or ketones in the presence of base would lead to various dipyrazolo[1,5-*a*:4',3'-*c*]pyridines **5**.

Thus, after these results, we decided to explore the one-pot tandem reaction with various 5-alkynylpyrazole-4-carbaldehydes **1**, *p*-toluenesulfonyl hydrazide and butyraldehyde (**4a**). As alkynyl functions, phenylethynyl (**1a**,**b**), 3-thienylethynyl (**1c**,**d**) and hex-1-ynyl (**1e**,**f**) were employed; silver triflate was used as the catalyst and K_3_PO_4_ as a base, needed for the formation of the second pyrazole ring. In this way, the dipyrazolo[1,5-*a*:4',3'-*c*]pyridines **5a**–**f** were achieved in yields of 44–83% (Table 1, entries 1–6). Replacement of butyraldehyde by propionaldehyde (**4b**) or 3-phenylpropanal (**4c**) afforded the corresponding tricyclic products **5g**, **5h** and **5i** in 59–73% yield, respectively (Table 1, entries 7–9). Lastly, application of the cyclic ketones cyclopentanone (**4d**), cyclohexanone (**4e**) and 2-methylcyclohexanone (**4f**) as carbonyl components resulted in the formation of tetracycles **5j**–**l** in acceptable yields (Table 1, entries 10–12).

**Table 1:** Multicomponent reaction of various 5-alkynyl-1-phenyl-1*H*-pyrazole-4-carbaldehydes **1** with *p*-toluenesulfonyl hydrazide and aldehydes or ketones **4**.

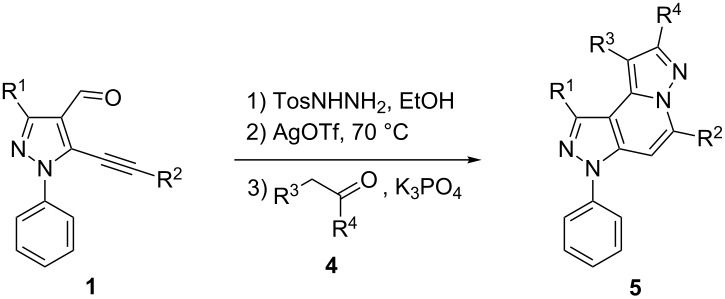				

Entry	Compound **1**	Compound **4**	Product **5**	Yield, %

1	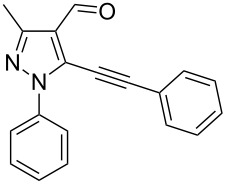 **1a**	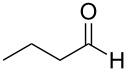 **4a**	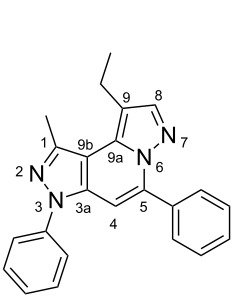 **5a**	83
2	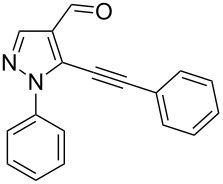 **1b**	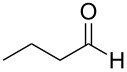 **4a**	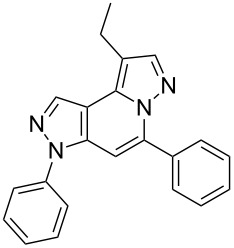 **5b**	47
3	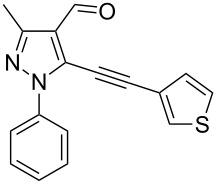 **1c**	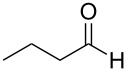 **4a**	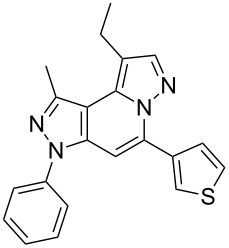 **5c**	73
4	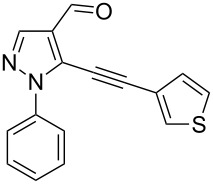 **1d**	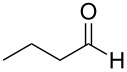 **4a**	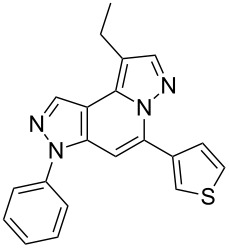 **5d**	73
5	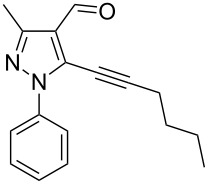 **1e**	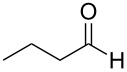 **4a**	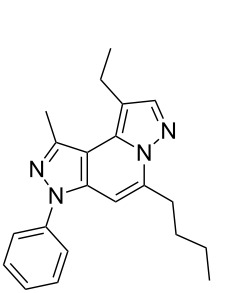 **5e**	44
6	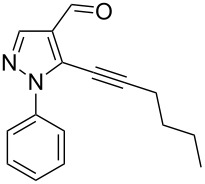 **1f**	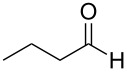 **4a**	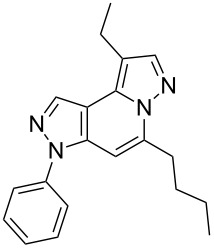 **5f**	79
7	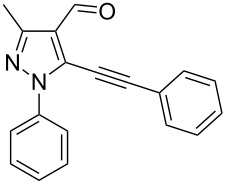 **1a**	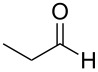 **4b**	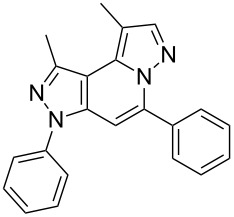 **5g**	59
8	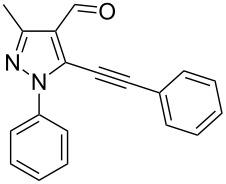 **1a**	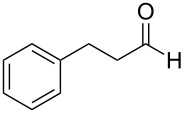 **4c**	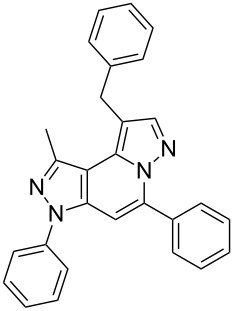 **5h**	73
9	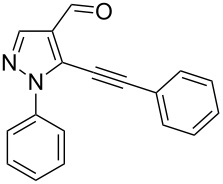 **1b**	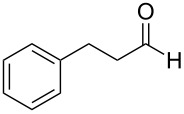 **4c**	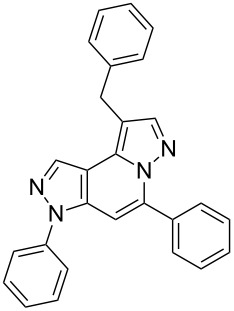 **5i**	59
10	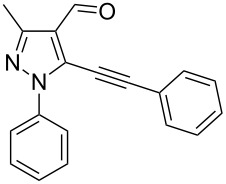 **1a**	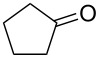 **4d**	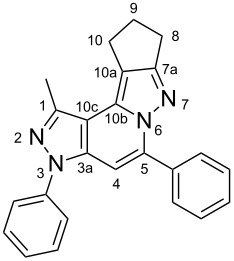 **5j**	46
11	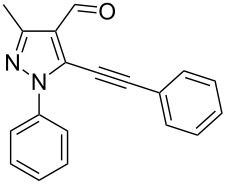 **1a**	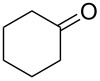 **4e**	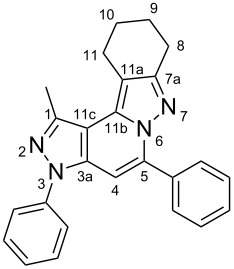 **5k**	64
12	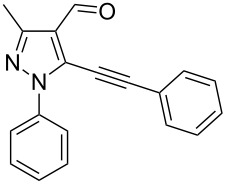 **1a**	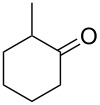 **4f**	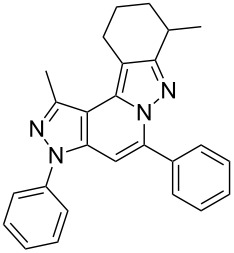 **5l**	38

### NMR spectroscopic investigations

The NMR spectroscopic data of all compounds described in this study are given in Supporting Information File 1. Unequivocal assignment of resonances was carried out by the combined application of various standard NMR spectroscopic techniques, such as ^1^H coupled ^13^C NMR spectra, APT, HMQC, gs-HSQC, gs-HMBC, COSY, TOCSY, NOESY and NOE-difference spectroscopy [29]. In some cases experiments with selective excitation of certain ^1^H resonances were performed, such as long-range INEPT [30] and 2D(δ,*J*) long-range INEPT [31], the latter experiments having been used for the unambiguous determination of long-range ^13^C,^1^H coupling constants.

With compound **2a**, the tosylhydrazone of starting aldehyde **1a**, (*E*)-configuration at the C=N double bond follows from an NOE between the iminyl-H (7.96 ppm) and NH (8.22 ppm) as well from the size of ^1^*J*(N=C–H) (160.7 Hz). In contrast, the (*Z*)-configuration and thus cis-position of the lone pair of the nitrogen and the coupled proton with respect to the C=N double bond should result in a considerably larger ^1^*J*-coupling due to lone-pair effects, which strongly influence such spin couplings [32–33].

**Figure 1:**
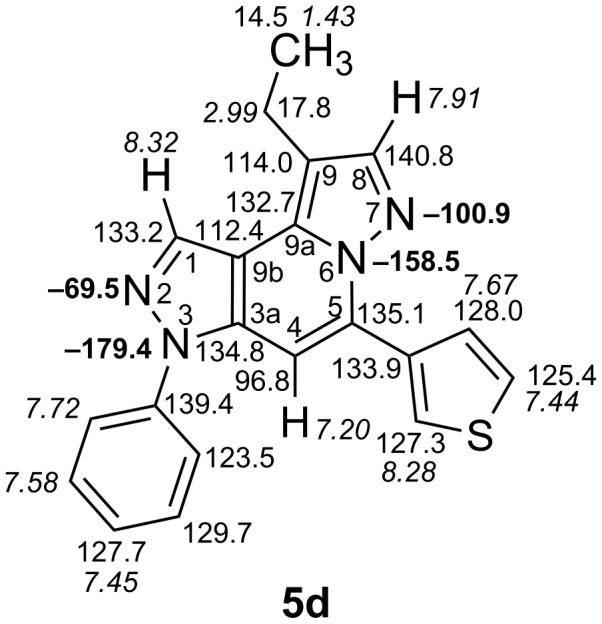
^1^H NMR (in italics), ^13^C NMR and ^15^N NMR (in bold) chemical shifts of **5d** in CDCl_3_ solution (with numbering of ring atoms).

The target products **5a**–**l** show very consistent signal sets regarding the invariable part of the molecules. In the ^1^H NMR spectra of congeners unsubstituted at position 1 (**5b**, **5d**, **5f**, **5i**) the signals due to H-1 and H-4 are split due to a small long-range coupling (^5^*J*(H-1,H-4) ~ 0.8 Hz). The signal of H-4 is located within a relatively small range, namely between 6.85 and 7.20 ppm. Those compounds unsubstituted at position 8 show the H-8 signal at 7.75–7.91 ppm. Characteristic core signals in the ^13^C NMR spectra are those of C-3a (134.8–136.7 ppm), C-4 (95.1–98.3 ppm), C-9 (**5j**: C-10a, **5k**,**l**: C-11a) (105.6–114.6 ppm), C-9a (**5j**: C-10b, **5k**,**l**: C-11b) (130.4–134.2) and C-9b (**5j**: C-10c, **5k**,**l**: C-11c) (111.2–112.7 ppm). The other carbon resonances (C-1, C-5, C-8) are influenced by the attached substituents to a somewhat larger extent. Also the ^15^N NMR spectra show a uniform pattern: the resonances of N-2 and N-3 are slightly influenced by the substituent at position 1 with the 1-H derivatives having slightly larger chemical shifts than those of the corresponding 1-methyl congeners. The ^15^N chemical shifts of N-6 and N-7 are somewhat smaller with derivatives **5j**–**5l** having a cycloaliphatic ring anellated to the concerning pyrazole ring. In Figure 1 the ^1^H, ^13^C and ^15^N NMR chemical shifts are displayed for model compound **5d**, for which also the numbering of ring atoms is given*.*

The obtained NMR spectroscopic data are inasmuch valuable as the availability of reliable, unambiguously assigned chemical shift data is important as reference material for NMR prediction programs, such as CSEARCH/NMRPREDICT [34–35] or ACD/C+H predictor [36]. Such programs have become more and more popular in the past few years, particularly for the prediction of ^13^C NMR chemical shifts. However, the quality of such predictions is highly dependent on the disposability of authentic reference data of related structures, a criterion which is frequently not fulfilled for rare or new condensed heterocyclic systems, such as those described here.

## Conclusion

In conclusion, we present a simple and nonlaborious method to access dipyrazolo[1,5-*a*:4',3'-*c*]pyridines from easily obtainable 5-alkynyl-1-phenyl-1*H*-pyrazole-4-carbaldehydes through a silver triflate catalyzed one-pot tandem reaction with *p*-toluenesulfonyl hydrazide and an appropriate aldehyde or ketone. Moreover, the novel heterocyclic system was investigated in great detail by extensive NMR spectroscopic investigations including also ^15^N. Further studies to exploit the synthetic potential as well as the biological activities of the latter compounds are in progress and will be published elsewhere.

## Supporting Information

File 1 Experimental details and characterization data.
