# Supplementary material for: Dipyrazolo[1,5-a:4',3'-c]pyridines – a new heterocyclic system accessed via multicomponent reaction
Source: Beilstein J Org Chem. 2012 Dec 27;8:2223–9. doi: 10.3762/bjoc.8.251 (PMC3557119; doi:10.3762/bjoc.8.251)
Supplement: File 1 — Experimental details and characterization data. [file Beilstein_J_Org_Chem-08-2223-s001.pdf]

Supporting Information

for

Dipyrazolo[1,5-*a*:4',3'-*c*]pyridines – a new heterocyclic system accessed via multicomponent reaction

Wolfgang Holzer*[1], Gytė Vilkauskaitė[1,2], Eglė Arbačiauskienė[2] and Algirdas Šačkus[2]

Address: [1] Department of Drug and Natural Product Synthesis, Faculty of Life Sciences, University of Vienna, Althanstrasse 14, A-1090 Vienna, Austria and [2]Institute of Synthetic Chemistry, Kaunas University of Technology, Radvilėnų pl. 19, 50254 Kaunas, Lithuania

Email: Wolfgang Holzer - wolfgang.holzer@univie.ac.at

* Corresponding author

Experimental details and characterization data

General

Melting points were determined on a Reichert–Kofler hot-stage microscope and are uncorrected. Mass spectra were obtained on a Shimadzu QP 1000 instrument (EI, 70 eV). Elemental analyses (C, H, N) were performed with an Exeter Analytical CE-440 Elemental Analyzer at the Microanalytical Laboratory, Kaunas University of Technology, and were in good agreement (±0.4%) with the calculated values. ^{1}H and ^{13}C NMR spectra were recorded on a Varian UnityPlus 300 spectrometer (299.95 MHz for ^{1}H, 75.43 MHz for ^{13}C) or on a Bruker Avance 500 spectrometer (500.13 MHz for ^{1}H,

125.77 MHz for ^{13}C) at 25 °C. The center of the solvent signal was used as an internal standard, which was related to TMS with δ 7.26 ppm (^{1}H in $CDCl_3$) and δ 77.0 ppm (^{13}C in $CDCl_3$). ^{15}N NMR spectra (50.68 MHz, referenced against external nitromethane) were obtained on a Bruker Avance 500 instrument with a ‘directly’ detecting broadband observe probe (BBFO). Digital resolutions were 0.25 Hz/data point in the ^{1}H spectra and 0.4 Hz/data point in the ^{13}C NMR spectra. Systematic names were generated with ACD/Name according to the IUPAC recommendations. For chromatographic separations, Kieselgel 60 (70–230 mesh, Merck) was used.

Synthetic procedures

Synthetic procedure for **2a**: 3-methyl-1-phenyl-5-(phenylethynyl)-1*H*-pyrazole-4-carbaldehyde (**1a**, 286 mg, 1 mmol) was added to a solution of *p*-toluenesulfonyl hydrazide (186 mg, 1 mmol) in ethanol (5 mL). The mixture was stirred at room temperature for 30 min. After completion of the reaction as indicated by TLC, the mixture was diluted with ethyl acetate (10 mL) and quenched with water (10 mL), extracted with ethyl acetate (3 × 10 mL). The organic layer was washed with brine, dried over Na_2SO_4 and concentrated under reduced pressure. The obtained residue was purified by flash chromatography on silica gel (eluent ethyl acetate/light petroleum, 1:4 v/v) to provide the desired product.

4-Methyl-N’-{(1E)-[3-methyl-1-phenyl-5-(phenylethynyl)-1H-pyrazol-4-yl]methylene}benzenesulfonohydrazide (**2a**).Yield: 440 mg (97%) of yellowish crystals; mp 79–80 °C. ^{1}H NMR (500 MHz, $CDCl_3$): δ = 2.40 (s, 3H, Tos-Me), 2.45 (s, 3H, $C\underline{H}_3$), 7.29 (m, 2H, SPh H-3,5), 7.32 (m, 2H, CPh H-3,5), 7.35 (m, 1H, NPh H-4), 7.36 (m, 1H, CPh H-4), 7.42 (m, 2H, CPh H-2,6), 7.45 (m, 2H, NPh H-3,5), 7.75 (m, 2H, NPh H-2,6), 7.89 (m, 2H, SPh H-2,6), 7.96 (s, 1H, CH), 8.22 (s, 1H, NH) ppm. ^{13}C NMR (125 MHz, $CDCl_3$): δ = 14.2 (3-Me, $^{1}J_{3\text{-Me}}$ = 128.9 Hz), 21.6 (Tos-Me, $^{1}J_{\text{Tos-Me}}$ = 127.2 Hz, $^{3}J_{\text{Tos-}\underline{C}\text{H3, Tos }\underline{H}\text{-3,5}}$ = 4.4 Hz), 76.6 ($\underline{C}$CPh), 100.7 (C$\underline{C}$Ph,), 119.0 (C-4, $^{2}J_{\text{C-4,NC}\underline{H}}$ = 7.1 Hz, $^{3}J_{\text{C-4,3-Me}}$ = 2.8 Hz), 121.4 (CPh C-1), 123.0 (NPh C-2,6), 125.9 (C-5, $^{3}J_{\text{C-5,NC}\underline{H}}$ = 2.5 Hz), 127.7 (NPh C-4), 128.1 (SPh C-2,6), 128.5 (CPh C-3,5), 128.9 (NPh C-3,5), 129.6 (SPh C-3,5), 131.5 (CPh C-2,6), 135.1 (SPh C-1), 139.2 (NPh C-1), 141.5 (N$\underline{C}$H, $^{1}J_{\text{N}\underline{C}\text{H, NC}\underline{H}}$ = 160.7 Hz, $^{2}J_{\text{N}\underline{C}\text{H,NN}\underline{H}}$ = 4.8 Hz), 144.2 (SPh C-4), 148.7 (C-3, $^{2}J_{\text{C-3,3-Me}}$ = 7.0 Hz, $^{3}J_{\text{C-3,NC}\underline{H}}$ = 5.3 Hz) ppm. ^{15}N NMR (50 MHz, $CDCl_3$): δ = −212.9 (NH), −166.6 (N-1), −75.4 (N-2), −69.9 (N) ppm. MS *m/z* (%): 454 (M^+, 4), 299 (30), 285 (52), 284 (41), 91 (94), 83 (23), 81 (21), 77 (100), 71 (35), 69 (45), 67 (23), 65 (65), 63 (30), 57 (79), 55 (63), 51 (66), 50

(21), 44 (62), 43 (72), 42 (21), 41 (47). Calcd. for $C_{26}H_{22}N_4O_2S$ (454.54)•0.5 H_2O: C, 67.37; H, 5.00; N, 12.09. Found: C, 67.04; H, 4.89; N, 12.12.

Synthetic procedure for **3a**: 3-methyl-1-phenyl-5-(phenylethynyl)-1*H*-pyrazole-4-carbaldehyde (**1a**, 286 mg, 1 mmol) was added to a solution of *p*-toluenesulfonyl hydrazide (186 mg, 1 mmol) in ethanol (5 mL). The mixture was stirred at room temperature for 30 min. Then AgOTf (26 mg, 10 mol %) was added and the reaction mixture was heated to 70 °C. After completion of the reaction as indicated by TLC, the mixture was diluted with ethyl acetate (10 mL), quenched with water (10 mL) and extracted with ethyl acetate (3 × 10 mL). The organic layer was washed with brine, dried over Na_2SO_4 and concentrated under reduced pressure. The obtained residue was purified by flash chromatography on silica gel (eluent ethyl acetate→dichloromethane/methanol, 9:1 v/v) to provide the desired product.

(3-Methyl-1,6-diphenyl-1H-pyrazolo[4,3-c]pyridine-5-ium-5-yl)(tosyl)amide (**3a**). Yield: 413 mg (91%) of yellowish crystals; mp 116–118 °C. ^{1}H NMR (500 MHz, $CDCl_3$): δ = 2.27 (s, 3H, PhC<u>H</u>$_3$), 2.77 (s, 3H, CC<u>H</u>$_3$), 6.78 (m, 2H, SPh H-3,5), 7.01 (m, 2H, SPh H-2,6), 7.19 (m, 2H, CPh H-3,5), 7.23 (m, 2H, CPh H-2,6), 7.31 (m, 1H, CPh H-4), 7.44 (m, 1H, NPh H-4), 7.49 (d, *J* = 0.6 Hz, 1H, H-7), 7.54 (m, 2H, NPh H-3,5), 7.61 (m, 2H, NPh H-2,6), 9.48 (d, *J* = 0.6 Hz, 1H, H-4) ppm. ^{13}C NMR (125 MHz, $CDCl_3$): δ = 12.2 (3-Me, $^1J_{3\text{-Me}}$ = 129.7 Hz), 21.2 (Tos-Me, $^1J_{\text{Tos-Me}}$ = 126.6 Hz, $^3J_{\text{Tos-}\underline{C}\text{H3, Tos }\underline{H}\text{-3,5}}$ = 4.4 Hz), 108.0 (C-7, $^1J_{\text{C-7,H-7}}$ = 172.9 Hz), 121.2 (C-3a, $^2J_{\text{C-3a,H-4}}$ = 4.6 Hz, $^3J_{\text{C-9a,H-7}}$ = 5.7 Hz, $^3J_{\text{C-3a,3-Me}}$ = 3.1 Hz), 122.7 (NPh C-2,6), 126.4 (SPh C-2,6), 127.5 (CPh C-3,5), 128.6 (NPh C-4), 128.8 (SPh C-3,5), 129.2 (CPh C-4), 130.0 (CPh C-2,6), 132.5 (CPh C-1), 137.7 (NPh C-1), 139.7 (SPh C-1), 140.2 (SPh C-4), 140.7 (C-7a), 145.5 (C-4, $^1J_{\text{C-4,H-4}}$ = 193.6 Hz), 147.1 (C-3, $^2J_{\text{C-3,3-Me}}$ = 7.2 Hz, $^3J_{\text{C-3,H-4}}$ = 2.0 Hz), 151.6 (C-6) ppm. ^{15}N NMR (50 MHz, $CDCl_3$): δ = −185.3 (N-1), −161.7 (N-5), −54.5 (N-2) ppm. MS *m/z* (%): 454 (M^+, 4), 299 (58), 298 (38), 285 (41), 284 (34), 105 (20), 91 (75), 77 (100), 65 (51), 63 (21), 51 (53). Calcd. for $C_{26}H_{22}N_4O_2S$ (454.54): C, 67.37; H, 5.00; N, 12.09. Found: C, 68.54; H, 4.73; N, 12.65.

General procedure for the synthesis of dipyrazolo[1,5-*a*:4,3-*c*]pyridines **5** by MCR:
The appropriate 5-alkynyl-1-phenyl-1*H*-pyrazole-4-carbaldehyde **1** (286 mg, 1 mmol) was added to a solution of *p*-toluenesulfonyl hydrazide (186 mg, 1 mmol) in ethanol (10 mL). The mixture was stirred at room temperature for 30 min. Then AgOTf (26 mg, 10 mol %) was added and the reaction mixture was heated to 70 °C. Subsequently, ketone or aldehyde **4** (2 mmol) and K_3PO_4 (636 mg, 3 mmol) were added in the mixture. After completion of the reaction as indicated by TLC, the mixture was diluted with ethyl

acetate (10 mL) and quenched with water (15 mL), extracted with ethyl acetate (3 × 15 mL). The organic layer was washed with brine, dried over Na_2SO_4 and concentrated under reduced pressure. The obtained residue was purified by flash chromatography on silica gel (eluent ethyl acetate/light petroleum, 1:6 v/v) to provide the desired product.

9-Ethyl-1-metyl-3,5-diphenyl-3H-dipyrazolo[1,5-a:4',3'-c]pyridine (**5a**). Yield: 293 mg (83%) of colorless crystals; mp 156–158 °C. ^{1}H NMR (500 MHz, $CDCl_3$): δ = 1.42 (t, J = 7.5 Hz, 3H, $CH_2C\underline{H}_3$), 2.91 (s, 3H, $C\underline{H}_3$), 3.12 (q, J = 7.5 Hz, 3H, $C\underline{H}_2CH_3$), 6.96 (s, 1H, H-4), 7.40 (m, 1H, NPh H-4), 7.49 (m, 3H, CPh H-3,4,5), 7.54 (m, 2H, NPh H-3,5), 7.66 (m, 2H, NPh H-2,6), 7.77 (m, 2H, CPh H-2,6), 7.86 (s, 1H, H-8) ppm. ^{13}C NMR (125 MHz, $CDCl_3$): δ = 15.7 (CH_3, $^{1}J_{CH3}$ = 126.5 Hz, $^{2}J_{\underline{C}H3,C\underline{H}2}$ = 4.8 Hz), 15.8 (1-Me, $^{1}J_{1\text{-}Me}$ = 128.2 Hz), 18.7 (CH_2, $^{1}J_{CH2}$ = 126.7 Hz, $^{2}J_{\underline{C}H2,C\underline{H}3}$ = 4.4 Hz), 98.1 (C-4, $^{1}J_{C\text{-}4,H\text{-}4}$ = 169.7 Hz), 111.7 (C-9b, $^{3}J_{C\text{-}9b,H\text{-}4}$ = 5.2 Hz, $^{3}J_{C\text{-}9b,1\text{-}Me}$ = 3.0 Hz), 113.4 (C-9, $^{2}J_{C\text{-}9,H\text{-}8}$ = 9.5 Hz), 123.6 (NPh C-2,6), 127.5 (NPh C-4), 128.3 (CPh C-3,5), 129.37 (CPh C-4), 129.40 (CPh C-2,6), 129.5 (NPh C-3,5), 133.3 (C-9a, $^{3}J_{C\text{-}9a,H\text{-}8}$ = 4.2 Hz, $^{3}J_{C\text{-}9a,C\underline{H}2}$ = 4.2 Hz), 134.4 (CPh C-1, $^{3}J_{CPh\ C\text{-}1,H\text{-}4}$ = 3.7 Hz), 136.5 (C-3a), 139.2 (NPh C-1), 140.1 (C-5, $^{2}J_{C\text{-}5,H\text{-}4}$ = 1.8 Hz), 141.7 (C-8, $^{1}J_{C\text{-}8,H\text{-}8}$ = 181.8 Hz, $^{3}J_{C\text{-}8,C\underline{H}2}$ = 4.8 Hz), 142.8 (C-1, $^{2}J_{C\text{-}1,1\text{-}Me}$ = 6.8 Hz) ppm. ^{15}N NMR (50 MHz, $CDCl_3$): δ = −184.7 (N-3), −156.2 (N-6), −101.4 (N-7), −74.3 (N-2) ppm. MS *m/z* (%): 352 (M^+, 49), 338 (24), 337 (100), 77 (22). Calcd. for $C_{23}H_{20}N_4$ (352.43): C, 78.38; H, 5.72; N, 15.90. Found: C, 78.19; H, 5.96; N, 16.03.

9-Ethyl-3,5-diphenyl-3H-dipyrazolo[1,5-a:4',3'-c]pyridine (**5b**). Yield: 159 mg (47%) of brownish crystals; mp 137–139 °C. ^{1}H NMR (500 MHz, $CDCl_3$): δ = 1.44 (t, J = 7.6 Hz, 3H, $CH_2C\underline{H}_3$), 2.99 (q, J = 7.6 Hz, 3H, $C\underline{H}_2CH_3$), 7.03 (d, J = 0.8 Hz, 1H, H-4), 7.43 (m, 1H, NPh H-4), 7.50 (m, 1H, CPh H-4), 7.51 (m, 2H, CPh H-3,5), 7.56 (m, 2H, NPh H-3,5), 7.72 (m, 2H, NPh H-2,6), 7.82 (m, 2H, CPh H-2,6), 7.88 (s, 1H, H-8), 8.34 (d, J = 0.8 Hz, 1H, H-1) ppm. ^{13}C NMR (125 MHz, $CDCl_3$): δ = 14.5 (CH_3, $^{1}J_{CH3}$ = 126.6 Hz, $^{2}J_{\underline{C}H3,C\underline{H}2}$ = 4.8 Hz), 17.8 (CH_2, $^{1}J_{CH2}$ = 126.8 Hz, $^{2}J_{\underline{C}H2,C\underline{H}3}$ = 4.4 Hz), 98.0 (C-4, $^{1}J_{C\text{-}4,H\text{-}4}$ = 169.7 Hz), 112.7 (C-9b, $^{2}J_{C\text{-}9b,H\text{-}1}$ = 10.5 Hz, $^{3}J_{C\text{-}9b,H\text{-}4}$ = 5.1 Hz), 114.0 (C-9, $^{2}J_{C\text{-}9,H\text{-}8}$ = 9.4 Hz, $^{2}J_{C\text{-}9,C\underline{H}2}$ = 6.3 Hz, $^{3}J_{C\text{-}9,C\underline{H}3}$ = 5.4 Hz), 123.4 (NPh C-2,6), 127.7 (NPh C-4), 128.4 (CPH C-3,5), 129.4 (CPH C-2,6), 129.5 (CPh C-4), 129.6 (NPh C-3,5), 132.6 (C-9a, $^{3}J_{C\text{-}9a,H\text{-}8}$ = 4.5 Hz, $^{3}J_{C\text{-}9a,C\underline{H}2}$ = 4.5 Hz), 133.3 (C-1, $^{1}J_{C\text{-}1,H\text{-}1}$ = 189.9 Hz), 134.1 (CPh C-1), 134.9 (C-3a, $^{3}J_{C\text{-}3a,H\text{-}1}$ = 3.8 Hz), 139.3 (NPh C-1), 140.2 (C-5), 141.0 (C-8, $^{1}J_{C\text{-}8,H\text{-}8}$ = 181.9 Hz, $^{3}J_{C\text{-}8,C\underline{H}2}$ = 4.7 Hz) ppm. ^{15}N NMR (50 MHz, $CDCl_3$): δ = −179.0 (N-3), −157.3 (N-6), −101.5 (N-7), −69.9 (N-2) ppm. MS *m/z* (%): 338 (M^+, 50), 332 (20), 324 (22), 323 (100), 317 (23), 290 (20), 275 (51), 271 (33), 77 (67), 51 (28). Calcd. for $C_{22}H_{18}N_4$ (338.41): C, 78.08; H, 5.36; N, 16.56. Found: C, 78.23; H, 5.62; N, 16.35.

9-Ethyl-1-methyl-3-phenyl-5-(3-thienyl)-3H-dipyrazolo[1,5-a:4',3'-c]pyridine (**5c**). Yield: 261 mg (73%) of brown crystals; mp 101–103 °C. ^{1}H NMR (500 MHz, $CDCl_3$): δ = 1.42 (t, J = 7.5 Hz, 3H, $CH_2C\underline{H}_3$), 2.88 (s, 3H, $C\underline{H}_3$), 3.10 (q, J = 7.5 Hz, 3H, $C\underline{H}_2CH_3$), 7.11 (s, 1H, H-4), 7.41 (m, 1H, NPh H-4), 7.41 (dd, $^3J_{\text{Th H-4,Th H-5}}$ = 5.0 Hz, $^4J_{\text{Th H-2,Th H-5}}$ = 3.1 Hz, 1H, Th H-5), 7.55 (m, 2H, NPh H-3,5), 7.64 (dd, $^3J_{\text{Th H-4,Th H-5}}$ = 5.0 Hz, $^4J_{\text{Th H-2,Th H-4}}$ = 1.2 Hz, 1H, Th H-4), 7.66 (m, 2H, NPh H-2,6), 7.89 (s, 1H, H-8), 8.19 (dd, $^4J_{\text{Th H-2,Th H-5}}$ = 3.1 Hz, $^4J_{\text{Th H-2,Th H-4}}$ = 1.2 Hz, 1H, Th H-2) ppm. ^{13}C NMR (125 MHz, $CDCl_3$): δ = 15.6 (CH_3, $^1J_{CH3}$ = 126.5 Hz, $^2J_{\underline{C}H3,C\underline{H}2}$ = 4.8 Hz), 15.8 (1-Me, $^1J_{\text{1-Me}}$ = 128.2 Hz), 18.6 (CH_2, $^1J_{CH2}$ = 127.1 Hz, $^2J_{\underline{C}H2,C\underline{H}3}$ = 4.5 Hz), 97.0 (C-4, $^1J_{\text{C-4,H-4}}$ = 169.1 Hz), 111.5 (C-9b, $^3J_{\text{C-9b,H-4}}$ = 5.1 Hz, $^3J_{\text{C-9b,1-Me}}$ = 3.1 Hz), 113.4 (C-9, $^2J_{\text{C-9,H-8}}$ = 9.6 Hz), 123.6 (Ph C-2,6), 125.2 (Th C-5, $^1J_{\text{Th C-5,Th H-5}}$ = 186.5 Hz, $^2J_{\text{Th C-5,Th H-4}}$ = 7.3 Hz, $^3J_{\text{Th C-5,Th H-2}}$ = 5.9 Hz), 127.1 (Th C-2, $^1J_{\text{Th C-2,Th H-2}}$ = 188.3 Hz, $^3J_{\text{Th C-2,Th H-4}}$ = 8.6 Hz, $^3J_{\text{Th C-2,Th H-5}}$ = 4.5 Hz), 127.4 (Ph C-4), 128.2 (Th C-4, $^1J_{\text{Th C-4,Th H-4}}$ = 169.7 Hz, $^2J_{\text{Th C-4,Th H-5}}$ = 5.1 Hz, $^3J_{\text{Th C-4,Th H-2}}$ = 8.6 Hz), 129.5 (Ph C-3,5), 133.3 (C-9a, $^3J_{\text{C-9a,H-8}}$ = 4.2 Hz, $^3J_{\text{C-9a,C}\underline{H}2}$ = 4.2 Hz), 134.1 (Th C-3, $^2J_{\text{Th C-3,Th H-2}}$ = 3.0 Hz, $^2J_{\text{Th C-3,Th H-4}}$ = 4.4 Hz, $^3J_{\text{Th C-3,Th H-5}}$ = 10.3 Hz, $^3J_{\text{Th C-3,H-4}}$ = 4.0 Hz), 135.1 (C-5, $^2J_{\text{C-5,H-4}}$ = 1.7 Hz, $^3J_{\text{C-5,Th H-2}}$ = 2.7 Hz), 139.2 (Ph C-1), 141.4 (C-8, $^1J_{\text{C-8,H-8}}$ = 181.7 Hz, $^3J_{\text{C-8,C}\underline{H}2}$ = 4.7 Hz), 142.8 (C-1, $^2J_{\text{C-1,1-Me}}$ = 6.9 Hz) ppm. ^{15}N NMR (50 MHz, $CDCl_3$): δ = −185.0 (N-3), −157.3 (N-6), −101.0 (N-7), −73.9 (N-2) ppm. MS *m/z* (%): 358 (M^+, 58), 343 (100), 77 (51), 57 (26), 51 (28), 43 (27). Calcd. for $C_{21}H_{18}N_4S$ (358.46): C, 70.36; H, 5.06; N, 15.63. Found: C, 70.32; H, 5.36; N, 15.60.

9-Ethyl-3-phenyl-5-(3-thienyl)-3H-dipyrazolo[1,5-a:4',3'-c]pyridine (**5d**). Yield: 251 mg (73%) of brownish crystals; mp 170–171 °C. ^{1}H NMR (500 MHz, $CDCl_3$): δ = 1.43 (t, J = 7.6 Hz, 3H, $CH_2C\underline{H}_3$), 2.99 (q, J = 7.6 Hz, 3H, $C\underline{H}_2CH_3$), 7.20 (d, J = 0.5 Hz, 1H, H-4), 7.44 (dd, $^3J_{\text{Th H-4,Th H-5}}$ = 5.1 Hz, $^4J_{\text{Th H-2,Th H-5}}$ = 3.1 Hz, 1H, Th H-5), 7.45 (m, 1H, NPh H-4), 7.58 (m, 2H, NPh H-3,5), 7.67 (dd, $^3J_{\text{Th H-4,Th H-5}}$ = 5.1 Hz, $^4J_{\text{Th H-2,Th H-4}}$ = 1.3 Hz, 1H, Th H-4), 7.72 (m, 2H, NPh H-2,6), 7.91 (s, 1H, H-8), 8.28 (dd, $^4J_{\text{Th H-2,Th H-5}}$ = 3.1 Hz, $^4J_{\text{Th H-2,Th H-4}}$ = 1.3 Hz, 1H, Th H-2), 8.32 (d, J = 0.9 Hz, 1H, H-1) ppm. ^{13}C NMR (125 MHz, $CDCl_3$): δ = 14.5 (CH_3, $^1J_{CH3}$ = 126.6 Hz, $^2J_{\underline{C}H3,C\underline{H}2}$ = 4.8 Hz), 17.8 (CH_2, $^1J_{CH2}$ = 126.8 Hz, $^2J_{\underline{C}H2,C\underline{H}3}$ = 4.4 Hz, $^3J_{\underline{C}H2,\text{H-8}}$ = 1.0 Hz), 96.8 (C-4, $^1J_{\text{C-4,H-4}}$ = 169.1 Hz), 112.4 (C-9b, $^2J_{\text{C-9b,H-1}}$ = 10.6 Hz, $^3J_{\text{C-9b,H-4}}$ = 5.2 Hz), 114.0 (C-9, $^2J_{\text{C-9,H-8}}$ = 9.5 Hz, $^2J_{\text{C-9,C}\underline{H}2}$ = 6.3 Hz, $^3J_{\text{C-9,C}\underline{H}3}$ = 5.4 Hz), 123.5 (Ph C-2,6), 125.4 (Th C-5, $^1J_{\text{Th C-5,Th H-5}}$ = 186.7 Hz, $^2J_{\text{Th C-5,Th H-4}}$ = 7.3 Hz, $^3J_{\text{Th C-5,Th H-2}}$ = 5.9 Hz), 127.3 (Th C-2, $^1J_{\text{Th C-2,Th H-2}}$ = 188.4 Hz, $^3J_{\text{Th C-2,Th H-4}}$ = 8.5 Hz, $^3J_{\text{Th C-2,Th H-5}}$ = 4.5 Hz), 127.7 (Ph C-4), 128.0 (Th C-4, $^1J_{\text{Th C-4,Th H-4}}$ = 169.9 Hz, $^2J_{\text{Th C-4,Th H-5}}$ = 5.1 Hz, $^3J_{\text{Th C-4,Th H-2}}$ = 8.6 Hz), 129.7 (Ph C-3,5), 132.7 (C-9a, $^3J_{\text{C-9a,H-8}}$ = 4.4 Hz, $^3J_{\text{C-9a,C}\underline{H}2}$ = 4.4 Hz), 133.2 (C-1, $^1J_{\text{C-1,H-1}}$ = 189.9 Hz), 133.9 (Th C-3, $^2J_{\text{Th C-3,Th H-2}}$ = 2.9 Hz, $^2J_{\text{Th C-3,Th H-4}}$ = 4.5 Hz, $^3J_{\text{Th C-3,Th H-5}}$ = 10.3 Hz, $^3J_{\text{Th C-3,H-4}}$ = 4.0 Hz), 134.8 (C-3a, $^3J_{\text{C-3a,H-1}}$ = 3.8 Hz), 135.1 (C-5), 139.4 (Ph C-1), 140.8 (C-8,

$^{1}J_{\text{C-8,H-8}}$ = 181.9 Hz, $^{3}J_{\text{C-8,C}\underline{\text{H}}\text{2}}$ = 4.6 Hz) ppm. ^{15}N NMR (50 MHz, $CDCl_3$): δ = −179.4 (N-3), −158.5 (N-6), −100.9 (N-7), −69.5 (N-2) ppm. MS *m/z* (%): 344 (M^+, 59), 330 (21), 329 (100), 77 (35). Calcd. for $C_{20}H_{16}N_4S$ (344.43): C, 69.74; H, 4.68; N, 16.27. Found: C, 69.75; H, 4.89; N, 16.44.

5-Butyl-9-ethyl-1-methyl-3-phenyl-3H-dipyrazolo[1,5-a:4',3'-c]pyridine (**5e**). Yield: 146 mg (44%) of yellowish crystals; mp 112–114 °C. ^{1}H NMR (500 MHz, $CDCl_3$): δ = 0.97 (t, *J* = 7.4 Hz, 3H, $CH_2CH_2C\underline{H}_3$), 1.40 (t, *J* = 7.5 Hz, 3H, $CH_2C\underline{H}_3$), 1.48 (m, 2H, $CH_2C\underline{H}_2CH_3$), 1.81 (m, 2H, $C\underline{H}_2CH_2CH_3$), 2.85 (s, 3H, $CC\underline{H}_3$), 3.08 (q, *J* = 7.5 Hz, 3H, $C\underline{H}_2CH_3$), 3.14 (m, 2H, $CC\underline{H}_2CH_2CH_3$), 6.79 (s, 1H, H-4), 7.41 (m, 1H, NPh H-4), 7.55 (m, 2H, NPh H-3,5), 7.64 (m, 2H, NPh H-2,6), 7.86 (s, 1H, H-8) ppm. ^{13}C NMR (125 MHz, $CDCl_3$): δ = 13.9 ($CH_2CH_2\underline{C}H_3$, $^{1}J_{\text{CH3}}$ = 124.8 Hz), 15.7 (CH_3, $^{1}J_{\text{CH3}}$ = 126.5 Hz, $^{2}J_{\underline{\text{C}}\text{H3,C}\underline{\text{H}}\text{2}}$ = 4.8 Hz), 15.8 (1-Me, $^{1}J_{\text{1-Me}}$ = 128.1 Hz), 18.7 (CH_2), 22.6 ($CH_2\underline{C}H_2CH_3$), 29.0 ($\underline{C}H_2CH_2CH_3$), 31.7 ($C\underline{C}H_2CH_2$), 95.1 (C-4, $^{1}J_{\text{C-4,H-4}}$ = 168.0 Hz, $^{3}J_{\text{C-4,C}\underline{\text{H}}\text{2}}$ = 4.8 Hz), 111.2 (C-9b, $^{3}J_{\text{C-9b,H-4}}$ = 5.1 Hz, $^{3}J_{\text{C-9b,1-Me}}$ = 2.9 Hz), 113.3 (C-9, $^{2}J_{\text{C-9,H-8}}$ = 9.4 Hz, $^{2}J_{\text{C-9,C}\underline{\text{H}}\text{2}}$ = 6.3 Hz, $^{3}J_{\text{C-9,C}\underline{\text{H}}\text{3}}$ = 5.3 Hz), 123.6 (Ph C-2,6), 127.3 (Ph C-4), 129.5 (Ph C-3,5), 132.8 (C-9a, $^{3}J_{\text{C-9a,H-8}}$ = 4.1 Hz, $^{3}J_{\text{C-9a,C}\underline{\text{H}}\text{2}}$ = 4.1 Hz), 136.5 (C-3a), 139.3 (Ph C-1), 141.2 (C-8, $^{1}J_{\text{C-8,H-8}}$ = 180.9 Hz, $^{3}J_{\text{C-8,C}\underline{\text{H}}\text{2}}$ = 4.7 Hz), 141.5 (C-5), 142.7 (C-1, $^{2}J_{\text{C-1,1-Me}}$ = 6.8 Hz) ppm. ^{15}N NMR (50 MHz, $CDCl_3$): δ = −180.3 (N-3), −155.1 (N-6), −104.0 (N-7), −71.9 (N-2) ppm. MS *m/z* (%): 332 (M^+, 38), 317 (38), 290 (41), 275 (100), 145 (34), 77 (40), 69 (37). Calcd. for $C_{21}H_{24}N_4$ (332.44): C, 75.87; H, 7.28; N, 16.85. Found: C, 76.14; H, 7.56; N, 16.81.

5-Butyl-9-ethyl-3-phenyl-3H-dipyrazolo[1,5-a:4',3'-c]pyridine (**5f**). Yield: 251 mg (79%) of yellowish crystals; mp 82–83 °C. ^{1}H NMR (500 MHz, $CDCl_3$): δ = 0.98 (t, *J* = 7.4 Hz, 3H, $CH_2CH_2C\underline{H}_3$), 1.41 (t, *J* = 7.6 Hz, 3H, $CH_2C\underline{H}_3$), 1.49 (m, 2H, $CH_2C\underline{H}_2CH_3$), 1.83 (m, 2H, $C\underline{H}_2CH_2CH_3$), 2.95 (q, *J* = 7.6 Hz, 3H, $C\underline{H}_2CH_3$), 3.16 (m, 2H, $CC\underline{H}_2CH_2CH_3$), 6.85 (d, *J* = 0.6 Hz, 1H, H-4), 7.44 (m, 1H, NPh H-4), 7.58 (m, 2H, NPh H-3,5), 7.69 (m, 2H, NPh H-2,6), 7.87 (s, 1H, H-8), 8.28 (d, *J* = 0.8 Hz, 1H, H-1) ppm. ^{13}C NMR (125 MHz, $CDCl_3$): δ = 13.9 ($CH_2CH_2\underline{C}H_3$, $^{1}J_{\text{CH3}}$ = 124.9 Hz), 14.5 (CH_3, $^{1}J_{\text{CH3}}$ = 126.6 Hz, $^{2}J_{\underline{\text{C}}\text{H3,C}\underline{\text{H}}\text{2}}$ = 4.9 Hz), 17.8 (CH_2), 22.6 ($CH_2\underline{C}H_2CH_3$), 29.0 ($\underline{C}H_2CH_2CH_3$), 31.5 ($C\underline{C}H_2CH_2$), 95.1 (C-4, $^{1}J_{\text{C-4,H-4}}$ = 168.1 Hz, $^{3}J_{\text{C-4,C}\underline{\text{H}}\text{2}}$ = 4.9 Hz), 112.1 (C-9b, $^{2}J_{\text{C-9b,H-1}}$ = 10.6 Hz, $^{3}J_{\text{C-9b,H-4}}$ = 5.1 Hz), 113.9 (C-9, $^{2}J_{\text{C-9,H-8}}$ = 9.4 Hz, $^{2}J_{\text{C-9,C}\underline{\text{H}}\text{2}}$ = 6.3 Hz, $^{3}J_{\text{C-9,C}\underline{\text{H}}\text{3}}$ = 5.4 Hz), 123.4 (Ph C-2,6), 127.6 (Ph C-4), 129.6 (Ph C-3,5), 132.1 (C-9a, $^{3}J_{\text{C-9a,H-8}}$ = 4.4 Hz, $^{3}J_{\text{C-9a,C}\underline{\text{H}}\text{2}}$ = 4.4 Hz), 133.2 (C-1, $^{1}J_{\text{C-1,H-1}}$ = 189.4 Hz), 134.9 (C-3a, $^{3}J_{\text{C-3a,H-1}}$ = 3.9 Hz), 139.5 (Ph C-1), 140.5 (C-8, $^{1}J_{\text{C-8,H-8}}$ = 181.1 Hz, $^{3}J_{\text{C-8,C}\underline{\text{H}}\text{2}}$ = 4.6 Hz), 141.7 (C-5) ppm. ^{15}N NMR (50 MHz, $CDCl_3$): δ = −180.3 (N-3), −155.1 (N-6), −104.0 (N-7), −71.9 (N-2) ppm. MS *m/z* (%): 318 (M^+, 36), 303 (31), 262 (21), 261 (100), 138 (29), 77 (37),

69 (22). Calcd. for $C_{20}H_{22}N_4$ (318.42): C, 75.44; H, 6.96; N, 17.60. Found: C, 75.59; H, 7.09; N, 17.21.

1,9-Dimethyl-3,5-diphenyl-3H-dipyrazolo[1,5-a:4',3'-c]pyridine (**5g**). Yield: 200 mg (59%) of colorless crystals; mp 187–189 °C. ^{1}H NMR (500 MHz, $CDCl_3$): δ = 2.64 (s, 3H, (C-9)C$\underline{H}_3$), 2.90 (s, 3H, (C-1)C$\underline{H}_3$), 6.96 (s, 1H, H-4), 7.40 (m, 1H, NPh H-4), 7.49 (m, 3H, CPh H-3,4,5), 7.53 (m, 2H, NPh H-3,5), 7.66 (m, 2H, NPh H-2,6), 7.78 (m, 2H, CPh H-3,5), 7.80 (s, 1H, H-8) ppm. ^{13}C NMR (125 MHz, $CDCl_3$): δ = 11.4 (9-Me, $^1J_{9\text{-Me}}$ = 127.3 Hz, $^3J_{9\text{-Me, H-8}}$ = 1.1 Hz), 16.0 (1-Me, $^1J_{1\text{-Me}}$ = 128.2 Hz), 98.0 (C-4, $^1J_{C\text{-}4,H\text{-}4}$ = 169.6 Hz), 106.0 (C-9, $^2J_{C\text{-}9,H\text{-}8}$ = 9.7 Hz, $^2J_{C\text{-}9,9\text{-Me}}$ = 6.4 Hz), 111.8 (C-9b, $^3J_{C\text{-}9b,H\text{-}4}$ = 5.2 Hz, $^3J_{C\text{-}9b,1\text{-Me}}$ = 3.0 Hz), 123.5 (NPh C-2,6), 127.4 (NPh C-4), 128.3 (CPh C-3,5), 129.4 (CPh C-2,4,6), 129.5 (NPh C-3,5), 134.0 (C-9a), 134.3 (CPh C-1), 136.3 (C-3a), 139.2 (NPh C-1), 140.1 (C-5), 142.9 (C-1, $^2J_{C\text{-}1,1\text{-Me}}$ = 6.9 Hz), 143.4 (C-8, $^1J_{C\text{-}8,H\text{-}8}$ = 181.9 Hz, $^3J_{C\text{-}8,9\text{-Me}}$ = 4.8 Hz) ppm. ^{15}N NMR (50 MHz, $CDCl_3$): δ = −184.7 (N-3), −155.8 (N-6), −102.0 (N-7), −74.2 (N-2) ppm. MS *m/z* (%): 339 (M + H^+, 24), 338 (M^+, 100), 337 (M − H^+, 85), 77 (49). Calcd. for $C_{22}H_{18}N_4$ (338.41): C, 78.08; H, 5.36; N, 16.56. Found: C, 77.85; H, 5.45; N, 16.57.

9-Benzyl-1-methyl-3,5-diphenyl-3H-dipyrazolo[1,5-a:4',3'-c]pyridine (**5h**). Yield: 302 mg (73%) of yellowish crystals; mp 110–112 °C. ^{1}H NMR (500 MHz, $CDCl_3$): δ = 2.70 (s, 3H, C$\underline{H}_3$), 4.50 (s, 2H, C$\underline{H}_2$), 7.01 (s, 1H, H-4), 7.22 (m, 1H, CH_2Ph H-4), 7.23 (m, 1H, CH_2Ph H-2,6), 7.31 (m, 1H, CH_2Ph H-3,5), 7.41 (m, 1H, NPh H-4), 7.51 (m, 3H, CPh H-3,4,5), 7.54 (m, 2H, NPh H-3,5), 7.66 (m, 2H, NPh H-2,6), 7.81 (m, 2H, CPh H-2,6), 7.75 (s, 1H, H-8) ppm. ^{13}C NMR (125 MHz, $CDCl_3$): δ = 15.6 (1-Me, $^1J_{1\text{-Me}}$ = 128.3 Hz), 31.0 (CH_2), 98.3 (C-4, $^1J_{C\text{-}4,H\text{-}4}$ = 169.8 Hz), 109.2 (C-9, $^2J_{C\text{-}9,H\text{-}8}$ = 9.5 Hz, $^2J_{C\text{-}9,CH2}$ = 6.9 Hz), 111.6 (C-9b), 123.7 (NPh C-2,6), 126.2 (CH_2Ph C-4), 127.6 (NPh C-4), 128.2 (CH_2Ph C-2,6), 128.4 (CPh C-3,5), 128.6 (CH_2Ph C-3,5), 129.5 (CPh C-2,4,6), 129.6 (NPh C-3,5), 134.2 (C-9a), 134.4 (CPh C-1, $^3J_{CPh\ C\text{-}1,H\text{-}4}$ = 3.7 Hz), 136.5 (C-3a), 139.1 (NPh C-1), 140.3 (C-5), 140.9 (CH_2Ph C-1), 143.0 (C-1, $^2J_{C\text{-}1,1\text{-Me}}$ = 6.9 Hz), 143.8 (C-8, $^1J_{C\text{-}8,H\text{-}8}$ = 182.9 Hz, $^3J_{C\text{-}8,C\underline{H}2}$ = 5.2 Hz) ppm. ^{15}N NMR (50 MHz, $CDCl_3$): δ = −184.5 (N-3), −155.8 (N-6), −100.5 (N-7), −74.0 (N-2) ppm. MS *m/z* (%): 414 (M^+, 15), 91 (26), 83 (20), 71 (46), 70 (20), 69 (40), 57 (94), 56 (26). 55 (63), 43 (100), 41 (53). Calcd. for $C_{28}H_{22}N_4$ (414.50)•0.1 H_2O: C, 80.78; H, 5.37; N, 13.46. Found: C, 81.19; H, 5.53; N, 13.07.

9-Benzyl-3,5-diphenyl-3H-dipyrazolo[1,5-a:4',3'-c]pyridine (**5i**). Yield: 236 mg (59%) of yellow oil. ^{1}H NMR (500 MHz, $CDCl_3$): δ = 4.34 (s, 2H, C$\underline{H}_2$), 7.06 (d, *J* = 0.6 Hz, 1H, H-4), 7.24 (m, 1H, CH_2Ph H-4), 7.33 (m, 4H, CH_2Ph H-2,3,56), 7.43 (m, 1H, NPh H-4),

7.51 (m, 1H, CPh H-4), 7.52 (m, 2H, CPh H-3,5), 7.55 (m, 2H, NPh H-3,5), 7.69 (m, 2H, NPh H-2,6), 7.84 (m, 2H, CPh H-2,6), 7.90 (s, 1H, H-8), 8.04 (d, *J* = 0.8 Hz, 1H, H-1) ppm. ^{13}C NMR (125 MHz, $CDCl_3$): δ = 30.6 (CH_2), 98.2 (C-4, $^1J_{C\text{-}4,H\text{-}4}$ = 169.9 Hz), 110.6 (C-9, $^2J_{C\text{-}9,H\text{-}8}$ = 9.5 Hz, $^2J_{C\text{-}9,CH2}$ = 7.0 Hz), 112.5 (C-9b, $^2J_{C\text{-}9b,H\text{-}1}$ = 10.6 Hz, $^3J_{C\text{-}9b,H\text{-}4}$ = 5.2 Hz), 123.4 (NPh C-2,6), 126.3 (CH_2Ph C-4), 127.7 (NPh C-4), 128.3 (CH_2Ph C-2,6), 128.4 (CPh C-3,5), 128.6 (CH_2Ph C-3,5), 129.4 (CPh C-2,6), 129.59 (NPh C-3,5), 129.6 (CPh C-4), 133.3 (C-9a), 133.6 (C-1, $^1J_{C\text{-}1,H\text{-}1}$ = 190.7 Hz), 133.9 (CPh C-1), 135.0 (C-3a, $^3J_{C\text{-}3a,H\text{-}1}$ = 3.8 Hz), 139.9 (NPh C-1, CH_2Ph C-1), 140.3 (C-5), 142.6 (C-8, $^1J_{C\text{-}8,H\text{-}8}$ = 182.9 Hz, $^3J_{C\text{-}8,C\underline{H}2}$ = 4.9 Hz) ppm. ^{15}N NMR (50 MHz, $CDCl_3$): δ = −179.2 (N-3), −156.8 (N-6), −101.3 (N-7), −69.9 (N-2) ppm. MS *m/z* (%): 400 (M^+, 0.25), 85 (20), 57 (24), 43 (100), 41 (31). Calcd. for $C_{27}H_{20}N_4$ (400.47): C, 80.98; H, 5.03; N, 13.99. Found: C, 81.18; H, 5.39; N, 13.67.

1-Methyl-3,5-diphenyl-3,9,10,11-tetrahydrocyclopenta[3,4]pyrazolo[1,5-a]pyrazolo[4,3-c]pyridine (**5j**). Yield: 168 mg (46%) of yellowish crystals; mp 181–182 °C. ^{1}H NMR (500 MHz, $CDCl_3$): δ = 2.58 (m, 2H, 2H-9), 2.78 (s, 3H, $C\underline{H}_3$), 2.96 (m, 2H, 2H-8), 3.17 (m, 2H, 2H-10), 6.87 (s, 1H, H-4), 7.37 (m, 1H, NPh H-4), 7.47 (m, 1H, CPh H-4), 7.49 (m, 2H, CPh H-3,5), 7.52 (m, 2H, NPh H-3,5), 7.68 (m, 2H, NPh H-2,6), 7.80 (m, 2H, CPh H-2,6) ppm. ^{13}C NMR (125 MHz, $CDCl_3$): δ = 13.6 (1-Me, $^1J_{1\text{-}Me}$ = 128.1 Hz), 24.8 (C-1), 25.4 (C-8), 29.8 (C-9), 96.8 (C-4, $^1J_{C\text{-}4,H\text{-}4}$ = 169.6 Hz), 111.5 (C-10c), 114.6 (C-10a), 123.1 (NPh C-2,6), 127.1 (NPh C-4), 128.4 (CPh C-3,5), 129.3 (CPh C-2,6), 129.5 (NPh C-3,5), 130.4 (C-10b), 134.7 (CPh C-1), 136.1 (C-3a), 139.5 (NPh C-1), 140.8 (C-5), 143.6 (C-1, $^2J_{C\text{-}1,1\text{-}Me}$ = 6.9 Hz), 164.8 (C-7a) ppm. ^{15}N NMR (50 MHz, $CDCl_3$): δ = −185.1 (N-3), −151.0 (N-6), −114.0 (N-7), −75.8 (N-2) ppm. MS *m/z* (%): 365 (M + H^+, 100), 364 (M^+, 25), 77 (45). Calcd. for $C_{24}H_{20}N_4$ (364.44): C, 79.10; H, 5.53; N, 15.37. Found: C, 79.13; H, 5.63; N, 15.40.

1-Methyl-3,5-diphenyl-8,9,10,11-tetrahydro-3H-pyrazolo[4',3':3,4]pyrido[1,2-b]indazole (**5k**). Yield: 242 mg (64%) of colorless crystals; mp 189–191 °C. ^{1}H NMR (500 MHz, $CDCl_3$): δ = 1.92 (m, 2H, 2H-9), 1.94 (m, 2H, 2H-10), 2.87 (s, 3H, $C\underline{H}_3$), 2.89 (t, *J* = 6.1 Hz, 2H, 2H-8), 3.16 (t, *J* = 6.0 Hz, 2H, 2H-11), 6.89 (s, 1H, H-4), 7.38 (m, 1H, NPh H-4), 7.47 (m, 1H, CPh H-4), 7.48 (m, 2H, CPh H-3,5), 7.52 (m, 2H, NPh H-3,5), 7.67 (m, 2H, NPh H-2,6), 7.82 (m, 2H, CPh H-2,6) ppm. ^{13}C NMR (125 MHz, $CDCl_3$): δ = 15.5 (1-Me, $^1J_{1\text{-}Me}$ = 128.2 Hz), 22.9 (C-11), 23.1 (C-9), 23.8 (C-10), 24.3 (C-8), 97.2 (C-4, $^1J_{C\text{-}4,H\text{-}4}$ = 169.5 Hz), 106.0 (C-11a), 111.7 (C-11c), 123.4 (NPh C-2,6), 127.2 (NPh C-4), 128.3 (CPh C-3,5), 129.45 (CPh C-2,6), 129.5 (NPh C-3,5), 133.3 (C-11b), 134.6 (CPh C-1), 136.4 (C-3a), 139.3 (NPh C-1), 139.9 (C-5), 143.0 (C-1, $^2J_{C\text{-}1,1\text{-}Me}$ = 1.7 Hz), 152.3 (C-7a) ppm. ^{15}N NMR (50 MHz, $CDCl_3$): δ = −185.3 (N-3), −160.5 (N-6),

−107.0 (N-7), −74.9 (N-2) ppm. MS *m/z* (%): 379 ($M + H^+$, 28), 378 (M^+, 100), 377 ($M - H^+$, 46), 350 (28), 349 (22), 77 (20). Calcd. for $C_{25}H_{22}N_4$ (378.47): C, 79.34; H, 5.86; N, 14.80. Found: C, 79.04; H, 5.95; N, 14.64.

1,8-Dimethyl-3,5-diphenyl-8,9,10,11-tetrahydro-3H-pyrazolo[4',3':3,4]pyrido[1,2-b]indazole (**5l**). Yield: 149 mg (38%) of yellow crystals; mp 178–180 °C. ^{1}H NMR (500 MHz, $CDCl_3$): δ = 1.41 (d, *J* = 6.9 Hz, 3H, $CHC\underline{H}_3$), 1.54 (m, 1H, H-9), 1.82 (m, 1H, H-10), 2.07 (m, 1H, H-9), 2.09 (m, 1H, H-10), 2.87 (s, 3H, $C\underline{H}_3$), 3.07 (m, 1H, H-8), 3.10 (m, 1H, H-11), 3.21 (m, 1H, H-11), 6.92 (s, 1H, H-4), 7.38 (m, 1H, NPh H-4), 7.465 (m, 1H, CPh H-4), 7.47 (m, 2H, CPh H-3,5), 7.53 (m, 2H, NPh H-3,5), 7.67 (m, 2H, NPh H-2,6), 7.89 (m, 2H, CPh H-2,6) ppm. ^{13}C NMR (125 MHz, $CDCl_3$): δ = 15.5 (1-Me, $^1J_{1\text{-Me}}$ = 128.1 Hz), 20.6 (8-Me), 22.4 (C-10), 23.1 (C-11), 29.8 (C-8), 32.1 (C-9), 97.1 (C-4, $^1J_{C\text{-}4,H\text{-}4}$ = 169.2 Hz), 105.6 (C-11a), 111.7 (C-11c, $^3J_{C\text{-}11c,H\text{-}4}$ = 5.2 Hz, $^3J_{C\text{-}11c,1\text{-Me}}$ = 3.1 Hz), 123.4 (NPh C-2,6), 127.2 (NPh C-4), 128.1 (CPh C-3,5), 129.2 (CPh C-4), 129.5 (NPh C-3,4), 129.6 (CPh C-2,6), 133.0 (C-11b), 134.5 (CPh C-1), 136.4 (C-3a), 139.3 (NPh C-1), 139.9 (C-5), 143.0 (C-1, $^2J_{C\text{-}1,1\text{-Me}}$ = 6.9 Hz), 156.6 (C-7a) ppm. ^{15}N NMR (50 MHz, $CDCl_3$): δ = −185.3 (N-3), −160.8 (N-6), −105.2 (N-7), −75.2 (N-2) ppm. MS *m/z* (%): 393 ($M + H^+$, 29), 392 (M^+, 100), 391 ($M - H^+$, 33), 364 (21), 363 (25). Calcd. for $C_{26}H_{24}N_4$ (392.50): C, 79.56; H, 6.16; N, 14.27. Found: C, 79.20; H, 6.37; N, 14.04.
